# Disproportion in Pericyte/Endothelial Cell Proliferation and Mechanisms of Intussusceptive Angiogenesis Participate in Bizarre Vessel Formation in Glioblastoma

**DOI:** 10.3390/cells10102625

**Published:** 2021-10-01

**Authors:** Lucio Díaz-Flores, Ricardo Gutiérrez, Miriam González-Gómez, María-del-Pino García, Lucio Díaz-Flores, Ibrahim González-Marrero, Julio Ávila, Pablo Martín-Vasallo

**Affiliations:** 1 Department of Basic Medical Sciences, Faculty of Medicine, University of La Laguna, 38071 Tenerife, Spain; kayto54@gmail.com (L.D.-F.); histologia54@gmail.com (R.G.); mirgon@ull.es (M.G.-G.); ldfvmri@yahoo.com (L.D.-F.J.); ibra.glez@gmail.com (I.G.-M.); 2 Instituto de Tecnologías Biomédicas de Canarias, University of La Laguna, 38071 Tenerife, Spain; javila@ull.edu.es; 3 Department of Pathology, Eurofins Megalab–Hospiten Hospitals, 38100 Tenerife, Spain; mpgarcias@mgalab.es; 4 Department of Bioquímica, Microbiología, Biología Celular y Genética, University of La Laguna, 38206 Tenerife, Spain

**Keywords:** glioblastoma, pericytes, endothelial cells, intussusceptive angiogenesis, bizarre/aberrant microvasculature, glomeruloid bodies, proliferation index

## Abstract

Glioblastoma (GBM) is the most malignant tumor in the brain. In addition to the vascular pattern with thin-walled vessels and findings of sprouting angiogenesis, GBM presents a bizarre microvasculature (BM) formed by vascular clusters, vascular garlands, and glomeruloid bodies. The mechanisms in BM morphogenesis are not well known. Our objective was to assess the role of pericyte/endothelial proliferation and intussusceptive angiogenic mechanisms in the formation of the BM. For this purpose, we studied specimens of 66 GBM cases using immunochemistry and confocal microscopy. In the BM, the results showed (a) transitional forms between the BM patterns, mostly with prominent pericytes covering all the abluminal endothelial cell (EC) surface of the vessels, (b) a proliferation index high in the prominent pericytes and low in ECs (47.85 times higher in pericytes than in ECs), (c) intravascular pillars (hallmark of intussusceptive angiogenesis) formed by transcapillary interendothelial bridges, endothelial contacts of opposite vessel walls, and vessel loops, and (d) the persistence of these findings in complex glomeruloid bodies. In conclusion, disproportion in pericyte/EC proliferation and mechanisms of intussusceptive angiogenesis participate in BM formation. The contributions have morphogenic and clinical interest since pericytes and intussusceptive angiogenesis can condition antiangiogenic therapy in GBM.

## 1. Introduction

Glioblastoma (GBM) is a highly malignant tumor with bizarre microvasculature (BM) [1,2,3,4]. The microvascular patterns in GBM include (a) thin-walled vessels with findings of microvascular sprouting (the “classic” vascular pattern), which is not the object of this study, (b) vascular clusters, (c) vascular garlands, and (d) glomeruloid bodies [1,2,3,4]. In this work, we focus our attention on cluster/garland-like/glomeruloid structures, also termed plexus, curled cell cords, and glomeruloid microvascular proliferations, which are the patterns that form the BM [1,5]. Glomeruloid bodies, the most complex and demonstrative of these bizarre vascular structures, are made up of closely packed anastomosing capillaries with irregular and tortuous, narrow lumina. Their main cell components are endothelial cells (ECs) and numerous pericytes [5,6,7]. Although glomeruloid bodies can be observed in other conditions in the brain (e.g., cancer metastasis) [8], they are an important finding for GBM diagnosis.

The mechanisms of high neovascularization in GBM have received great attention, including the presence of bizarre vessels and resistance to antiangiogenic therapy, which can be related to different types of angiogenesis [1,2,3,4,5,9,10,11,12,13,14,15,16,17]. It has been suggested that intussusception (intussusceptive microvascular growth) could be a mechanism of compensatory vascular growth occurring in human glioma [18,19], with advantages and inconveniences over classic sprouting angiogenesis [18]. Thus, sprouting and intussusceptive angiogenesis could be complementary mechanisms in human gliomas. In intussusceptive angiogenesis, the preexisting vasculature is split and remodeled by intravascular pillars, which are formed through several procedures, including endothelial bridges, merged adjacent capillaries with modified contacting walls, and vessel loops surrounding interstitial tissue structures (ITSs) [20,21,22,23,24,25,26,27]. Likewise, it has been highlighted that the prevalence and association of some of these intussusceptive mechanisms can determine the predominant pattern of pathological vessels [28,29]. The study of this possibility is of interest to explain the histogenesis of bizarre vessels in GBM, as well as therapy resistance due to a switch from sprouting to intussusceptive angiogenesis. EC proliferation in intussusceptive angiogenesis is low and compensated for by the spreading and thinning of ECs [24,30,31,32]. Therefore, it is also of interest to explore the overall proliferation index (PI) in BM and specifically in each cell type (EC and pericyte).

Given the above, the objective of this study was to assess the role of EC and pericyte proliferative activity and intussusceptive angiogenic mechanisms in the formation of the BM in GBM. To this end, in the BM, we explore (a) the main types and distribution of cells that comprise or are related to it, (b) the proliferative activity in its global components and in the ECs and pericytes separately, and (c) the findings supporting the participation of vessel intussusception (intussusceptive angiogenesis) in its morphogenesis.

## 2. Materials and Methods

### 2.1. Human Tissue Samples

The archives of Histology and Anatomical Pathology of the Departments of Basic Medical Sciences of La Laguna University, University Hospital, and Eurofins^®^ Megalab–Hospiten Hospitals of the Canary Islands were searched for cases of glioblastoma multiforme. Specimens (paraffin blocks) were obtained from 66 cases. Patients were Caucasian (38 males and 27 females, aged 17–88 years). All samples were studied by conventional histologic techniques. From them, 40 cases with more evident zones showing BM (including glomeruloid bodies) were used for immunochemistry procedures and immunofluorescence in confocal microscopy. Ethical approval for this study was obtained from the Ethics Committee of La Laguna University (Comité de Ética de la Investigación y de Bienestar Animal, CEIBA 2021-3069), including the dissociation of the samples from any information that could identify the patient. The authors, therefore, had no access to identifiable patient information.

### 2.2. Light Microscopy

Specimens for conventional light microscopy were fixed in a buffered neutral 4% formaldehyde solution, embedded in paraffin, and cut into 3 µm thick sections. Sections were stained with hematoxylin and eosin (H&E).

### 2.3. Immunohistochemistry

Two types of procedures were performed. In the first procedure (automated), 3 μm thick histologic sections were attached to silanized slides. The sections were placed in a 60 °C oven overnight and in a BOND-MAX Automated Immunohistochemistry Vision Biosystem (Leica Microsystems GmbH, Wetzlar, Germany). Deparaffinized sections were pretreated with Epitope Retrieval Solution 2 (EDTA buffer pH 8.8) at 98 °C for 20 min. Using the Bond Polymer Refine Detection Kit DC9800 (Leica Microsystems GmbH, Wetzlar, Germany), peroxidase blocking was carried out for 10 min after the washing steps. Newly washed, the tissues were incubated with anti-CD34 (Bond™ PA0212; Leica Biosystems, Newcastle, UK), anti-αSMA (Bond™ PA0943; Leica Biosystems, Newcastle, UK), Ki-67 (Bond™ PA0118; Leica Biosystems, Newcastle, UK), GFAP (glial fibrillary acidic protein) (Bond™ PA0026; Leica Biosystems, Newcastle, UK), calponin (Bond™ PA0416; Leica Biosystems, Newcastle, UK), muscle-specific actin (MSA) (Bond™ PA0258; Leica Biosystems, Newcastle, UK), Desmin (Bond™ PA0032), and Caldesmon (Bond™ MAD-005084QD-7) (VITRO, Sevilla, Spain) primary antibodies for 30 min, incubated with polymer for 10 min, and developed with DAB Chromogen for 10 min. For double immunostaining (CD34/αSMA, CD34/Ki-67, αSMA/Ki-67, GFAP/αSMA) a similar automated procedure was performed with the following modification: (a) Bond™ Polymer Refine Detection Kit chromogen DAB with the HRP enzyme which is visualized via a brown precipitate, and (b) Bond™ Polymer Refine Red Detection Kit chromogen Fast Red with the alkaline phosphatase (AP) enzyme, which is visualized via a red precipitate. In the second procedure (nonautomated), 3 μm thick histologic sections were attached to silanized slides. After rehydration, sections were boiled in 10 mM citrate buffer (pH 6) at 100 °C for 20 min for antigen retrieval, rinsed in Tris-buffered saline (TBS; pH 7.6, 0.05 M), blocked with 3% hydrogen peroxide, and then incubated with the following primary antibodies diluted in TBS overnight in a humid chamber at room temperature: rabbit polyclonal anti-CD34 (1/100 dilution, code no. A13929, AB clonal, Woburn, MA, USA) and mouse monoclonal anti αSMA (1/100 dilution, code no. ABK1-A8914, Abyntek Biopharma Vizcaya, Spain). The following day, sections were washed thrice in TBS and incubated for 1 h with biotinylated secondary goat anti-rabbit antibody (1/200, code no. OS03B, Calbiochem) or biotinylated secondary goat anti-mouse antibody (1/200, code no. 401213, Calbiochem), depending on the primary antibody. After several washes in TBS, sections were incubated for 1 h with streptavidin–biotin peroxidase complex (1/200, code no. 189730, 2031941, Calbiochem, Merck KGaA, Darmstadt, Germany) and developed using a TBS solution containing 0.04% 3,3′-diaminobenzidine (DAB) and 0.01% hydrogen peroxide by 5 min immersion. Sections were then briefly counterstained with hematoxylin, dehydrated in ethanol series, cleared in xylene, and mounted in Eukitt^®^. Positive and negative controls were used. For the double immunostaining, we used anti-CD34 antibody (diaminobenzidine, DAB, as chromogen) to highlight CD34^+^ ECs and anti-αSMA (aminoethylcarbazole, AEC, substrate-chromogen) for pericytes.

### 2.4. Immunofluorescence in Confocal Microscopy

For immunofluorescence, 3 µm and 10 µm thick tissue sections were obtained as described above. For antigen retrieval, sections were deparaffinized and boiled for 20 min in sodium citrate buffer 10 mM (pH 6), rinsed in TBS (pH 7.6, 0.05 M), and incubated with the following primary antibodies diluted in TBS overnight in a humid chamber at room temperature: rabbit polyclonal anti-CD34 (1/100 dilution, code no. A13929, AB clonal), mouse monoclonal anti-CD34 (Ready to use, code no IR632, DAKO Glostrup, Denmark) anti-phospho-Histone H3 (Ser10) (mitosis marker, 1/300 dilution, code no. 06-570, Millipore), anti-GFAP (Ready to use, code no. IR524, DAKO Glostrup, Denmark), and anti-Iba1 (1/200 dilution, code no. 019-19741, FUJIFILM Wako), and mouse monoclonal anti-αSMA (1/100 dilution, code no. ABK1-A8914, Abyntek Biopharma). For double immunofluorescence labeling, sections were incubated combining each polyclonal antibody with the monoclonal one (anti-αSMA). The next day, the slides were rinsed in TBS and incubated for 1 h at room temperature in the dark with the secondary biotinylated goat anti-mouse IgG, H + L Chain specific Biotin conjugate (1:300, Calbiochem, cat. No. 401213, Calbiochem) and Alexa Fluor 488 goat anti-rabbit IgG (H + L) antibodies (1:300, cat. No. A11001, Invitrogen (Waltham, MA, USA), followed by incubation with streptavidin Cy3 conjugate (1:500, Code: SA1010, Invitrogen) for 1 h at room temperature in the dark. Nuclei were detected by DAPI staining (Chemicon International, Temecula, CA, USA). After being washed in TBS, sections were exposed to a saturated solution of Sudan black B (Merck, Barcelona, Spain) for 20 min to block autofluorescence. They were rinsed in TBS and cover-slipped with DABCO (1%) and glycerol–PBS (1:1). Negative controls were performed in the absence of primary antibodies. Fluorescence immunosignals were obtained using a Fluoview 1000 laser scanning confocal imaging system (Olympus Optical, Shinjuku, Tokyo, Japan).

### 2.5. Evaluation of the PI in the BM of GBM

Using the monoclonal antibody Ki-67, which reacts with a protein expressed in the cell-cycle phases G1, G2, S, and M, the PI (growth fraction) was evaluated in the BM of GBM using a high-power objective lens (40×, magnification 400) and immersion objective lens (100×, magnification 1000) and by two independent observers. When the entire nucleus or a part of it was observed to express Ki-67, the cell was considered positive, and the index was established depending on the proportion of positive cells in relation to the total cells evaluated. In addition to Ki-67, we used the PHH3 as a very specific way to detect proliferative activity, thereby complementing and confirming the results obtained with Ki-67. Mitoses were also observed, and the weak staining of the pericytes by muscle-specific actin (MSA) was very useful since, while it revealed the cell type, it did not hide the mitosis. To specifically calculate the PI in the ECs or pericytes of the BM, double immunochemistry for Ki-67 and CD34 (EC marker) or αSMA (pericyte marker) was performed, respectively. For the quantitative study of cells with or without nuclear Ki-67 expression, 200 nuclei of cells expressing their cytoplasmic marker were examined in the BM of each selected case. In addition, samples of two adjacent sections of the same block tumor with superimposed images were used to confirm the previously obtained data. Pearson’s chi-squared test was used for statistical analysis.

## 3. Results

### 3.1. Microvascular Patterns in GBM

**Figure 1:**
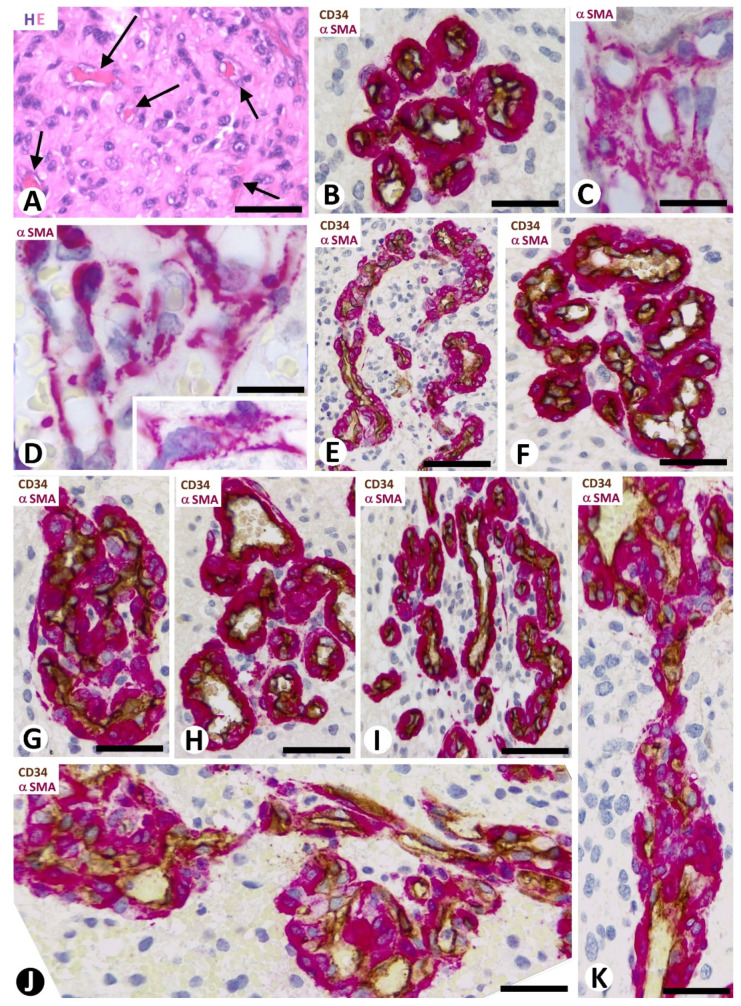
Vascular patterns and transitional structures in GBM. (**A**) “Classic” pattern with delicate capillaries (arrows). (**B**–**G**) Patterns of vascular clusters (**B**–**D**), vascular garlands (**E**), and glomeruloid bodies (**F**,**G**), which form the bizarre microvasculature (BM). In vascular clusters, note the different characteristics of pericytes (red) in Type I (**B**) or Type II (**C**,**D**) BM. (**H**,**I**) Transitional structures between vascular garlands and glomeruloid bodies. (**J**,**K**) Glomeruloid bodies are observed originating from vessels of varying size and transitional structures. (**A**) Hematoxylin staining. (**B**,**E**–**K**) Double immunochemistry for CD34 (brown, vascular endothelium) and αSMA (red, pericytes). Hematoxylin counterstain. (**C**,**D**) Immunochemistry for αSMA. Hematoxylin counterstain. Bars: (**A**) 150 µm, (**B**–**D**,**F**,**J**,**K**) 40 µm, and (**E**,**G**–**I**) 45 µm.

Except for vasculogenic mimicry, the major vascular patterns in GBM were a thin-walled microvasculature (Figure 1A) with findings of microvascular sprouting (“classic” vascular pattern), vascular clusters (Figure 1B–D), vascular garlands (Figure 1E), and glomeruloid bodies (Figure 1F,G), with the last three forming the BM. Transitional structures were observed between clusters and garland-like and glomeruloid structures (glomeruloid bodies and precursor structures) (Figure 1H,I). These structures were frequently joined by vessels of varying size (Figure 1J,K).

### 3.2. Cellular Components of Bizarre Vessels in GBM

**Figure 2:**
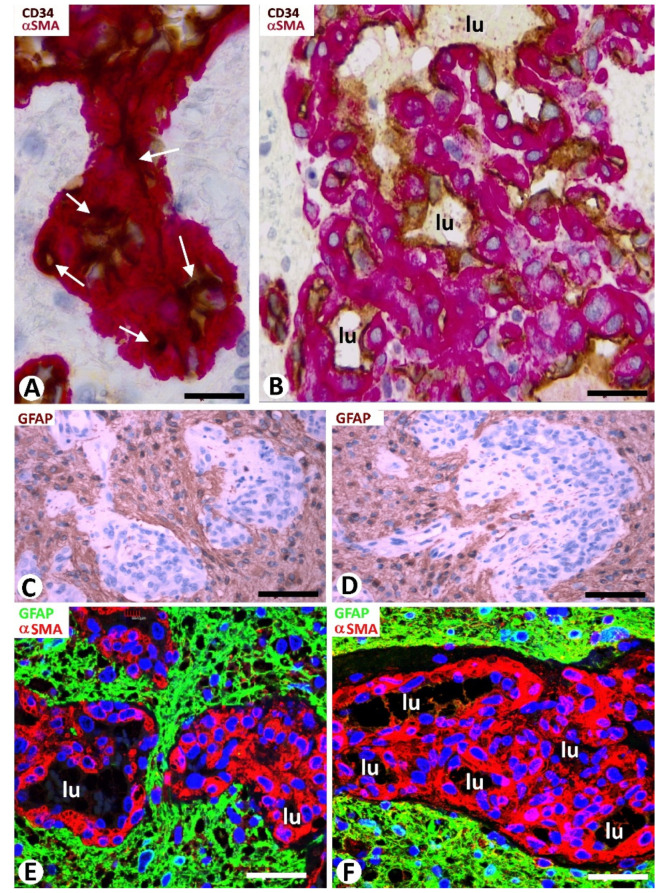
Cellular components of the bizarre vessels and their relationship with neoplastic cells in GBM. (**A**,**B**) αSMA^+^ pericytes (red), increased in number and size, are observed around ECs (brown) of vessels with virtual lumen (**A**, arrows) or with small lumen (**B**, lu in a glomeruloid body). (**C**–**F**) The neoplastic glial cells (brown in **C**,**D**; green in **E**,**F**) are observed around glomeruloid bodies (hematoxylin-stained nuclei in (**C**,**D**); αSMA-stained pericytes in (**E**,**F**). Vessel lumen: lu). Note rows of neoplastic glial cells separating glomeruloid bodies in (**C**,**E**). (**A**,**B**) Double immunochemistry for CD34 (brown) and αSMA (red). Hematoxylin counterstain. (**C**,**D**) Immunochemistry for GFAP (glial fibrillary acidic protein) (brown). Hematoxylin counterstain. (**E**,**F**) Double immunofluorescence for GFAP (green) and αSMA (red). DAPI counterstain. Bar: (**A**,**B**) 20 µm, (**C**,**D**) 45 µm, and (**E**,**F**) 40 µm.

ECs and pericytes were the two main types of cells in bizarre vessels in GBM. CD34 and αSMA were expressed in ECs and pericytes, respectively. H-caldesmon, desmin, and calponin only were expressed by pericytes in a few vessels. In most of the cluster/garland-like/glomeruli (Type I BM) (about 85% of BM), prominent pericytes showed a cell body increased in size, with a plump aspect. These prominent, plump αSMA^+^ pericytes covered all the abluminal EC surface of the vessels in the glomeruloid bodies and precursor structures (Figure 1E–K and Figure 2A,B). With relative frequency, the slit-like lumen of these vessels was virtual or narrow, and the confluent αSMA^+^ pericytes around the folded vessels appeared as aggregates (the flattened unstained EC cytoplasm was not detected), except when double immunochemistry for CD34 and αSMA was undertaken (Figure 2A,B).

In approximately 50% of the clusters and occasional vascular garlands (Type II BM) (about 15% of BM), pericytes partially covered the abluminal surface of ECs and presented an elongated cell body with long slender processes and an elaborate system of longitudinal and circumferential branches encircling the ECs (Figure 1C,D).

### 3.3. Neoplastic Glial Cells and BM in GBM

**Figure 3:**
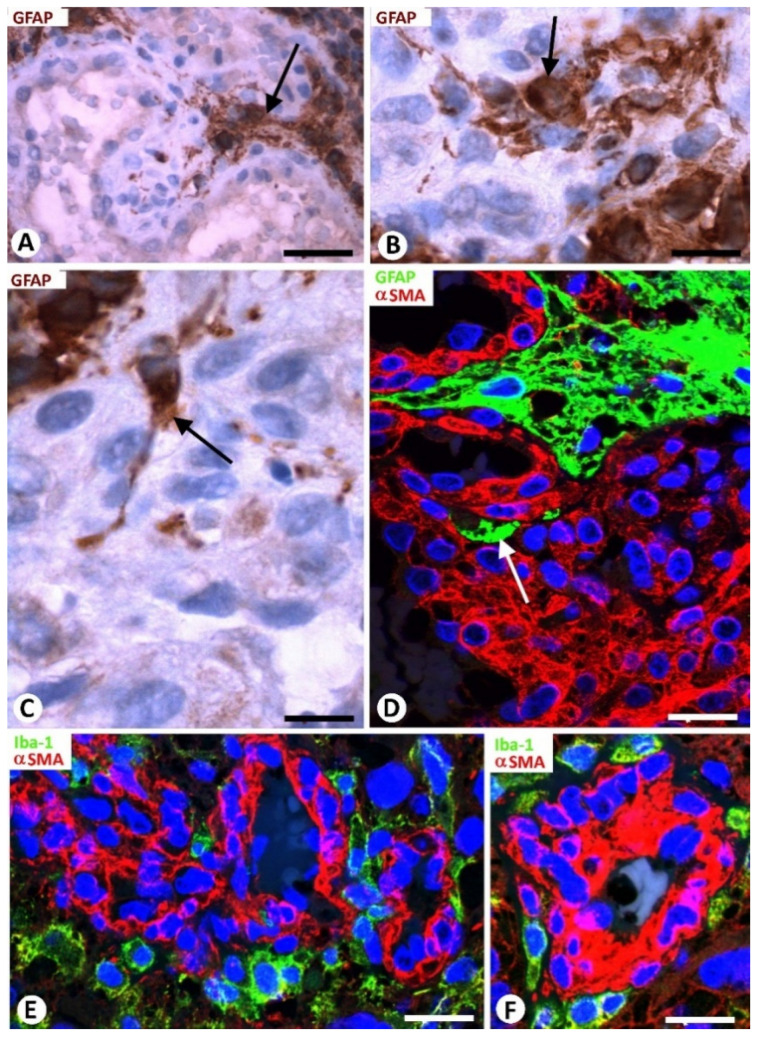
Relationship of neoplastic glial cells (**A**–**D**) and microglia (**E**,**F**) with bizarre vessels in GBM. (**A**–**D**) Images of occasional neoplastic glial cells (brown in (**A**–**C**); green in (**D**), arrows) incorporated between glomeruloid body components (hematoxylin-stained nuclei in (**A**–**C**) and αSMA+ pericytes (red) in (**D**)). (**E**,**F**) Microgliocytes with an ameboid aspect (green) are observed around pericytes (red) of bizarre vessels. (**A**–**C**) Immunochemistry for GFAP (brown). Hematoxylin counterstain. (**D**) Double immunofluorescence for GFAP (green) and αSMA (red). DAPI counterstain. (**E**,**F**) Double immunofluorescence for Iba1 (green) and αSMA (red). Bar: (**A**) 40 µm, (**B**,**C**) 15 µm, and (**E**,**F**) 30 µm.

Neoplastic glial cells expressing GFAP were observed around the BM in GBM (Figure 2C–F). This finding was highlighted by double immunochemistry (Figure 2C,D) and double immunofluorescence (Figure 2E,F) for GFAP and αSMA. Rows of neoplastic glial cells were seen separating neighboring glomeruloid bodies from each other or from their precursor structures (Figure 2E) and occasionally within them (Figure 3A–D).

### 3.4. Microglia and BM in GBM

Isolated Iba1^+^ microglial cells were observed in areas adjacent to the BM in GBM using double immunofluorescence for Iba1 and αSMA (Figure 3E,F). Microgliocytes showed an increased body, with an amoeboid aspect.

### 3.5. Pericyte and EC Proliferation in the BM

Different results in terms of the PI in pericytes and ECs were obtained for Type I BM, as well as between pericytes of both types of BM (Table 1).

**Figure 4:**
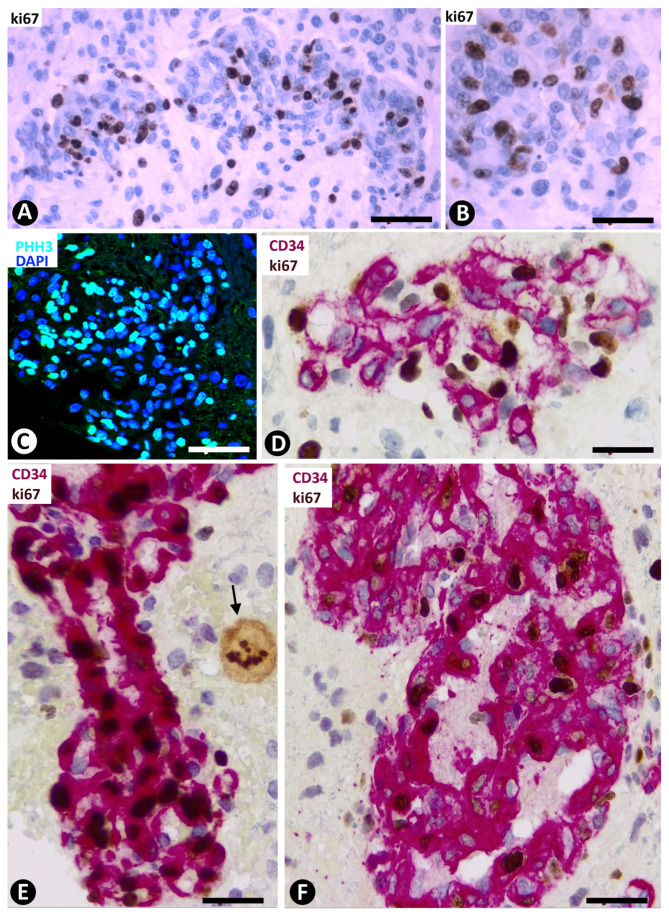
Cell proliferation in bizarre vessels in GBM. (**A**–**C**) Images showing high numbers of cell nuclei expressing Ki-67 (brown in **A**,**B**) and PHH3 (green in **C**) in glomeruloid bodies (high proliferation index). (**D**) CD34 ECs (red) do not show Ki-67 expression in their nuclei. Nuclei that express Ki-67 (brown) around ECs are compatible with unmarked pericytes (compare with Figure 4E,F). (**E**,**F**) Images showing pericytes (red) with high numbers of nuclei expressing Ki-67 (brown). Note in (**E**) a Ki67^+^ neoplastic cell in mitosis (arrow). (**A**,**B**) Immunochemistry for Ki-67. Hematoxylin counterstain. (**C**) Immunofluorescence for phospho-histone H3 (PHH3). DAPI counterstain. (**D**) Double immunochemistry for CD34 (red) and Ki-67 (brown). (**E**,**F**) Double immunochemistry for αSMA (red) and Ki-67 (brown). Hematoxylin counterstain. Bar: (**A**,**B**,**D**) 40 µm, (**C**) 45 µm, and (**E**,**F**) 30 µm.

**Figure 5:**
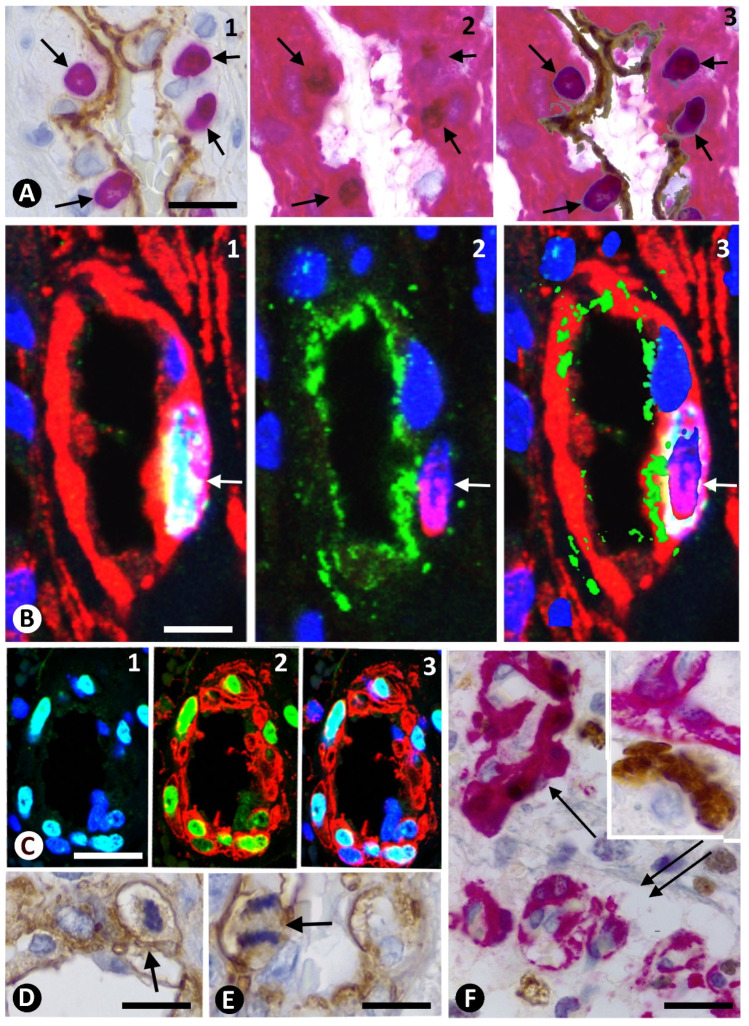
**(A1–3**,**B1–3**) The relative positions of ECs and pericytes are observed in inmmunochmistry (**A**) and inmmunofluoresence (**B**) in adjacent cuts (nuclei expressing Ki-67 in A and PHH3 in (**B**): arrows). (**A**) 1, CD34 (brown) and Ki-67 (red); 2, αSMA (red) and Ki-67 (brown); 3, merged. (**B**) 1, αSMA (red) and PHH3 (green); 2, CD34 (green) and PHH3 (red); 3, merged. DAPI counterstain. (**C1–3**) Images showing pericytes (red) with high numbers of nuclei expressing PHH3 (green). 1, PHH3 and DAPI; 2, αSMA and PHH3; 3, merged. (**D**,**E**) Mitosis (arrows) in MSA^+^ pericytes (brown). Immunochemistry for MSA, Hematoxylin counterstain. (**F**) Vessels of Type I and Type II BM. Note the nuclear expression of Ki-67 (brown) in several pericytes in a Type I BM vessel (arrow) and absence of Ki-67 nuclear expression in Type II BM vessels (double arrow). Insert: A Type II BM vessel with high magnification. Note a neoplastic cell expressing Ki-67. Bar: (**A**,**C**–**F**) 30 µm and (**B**) 10 µm.

In the BM, including all the cells in glomeruloid bodies and their precursor structures, the expression of Ki-67 and PHH3 was increased (Figure 4A–C). The percentage of nuclei expressing Ki-67, with respect to total nuclei (PI), was 43.57% ± 9.5% in Type I BM and 3.41% ± 0.76% in Type II BM (Table 1). In Type I BM, by double immunochemistry for CD34 and Ki-67 (Figure 4D), the PI in ECs was 0.88% ± 0.29%. Through double immunochemistry for αSMA and Ki-67 (Figure 4E,F), the PI in pericytes was 42.11% ± 5.76%. Therefore, the relationship between the PI of pericytes and ECs in Type I BM showed that, in pericytes, it was 47.85 times higher than in ECs. Samples showing the relative positions of ECs and pericytes by superimposed images of two adjacent sections of the same block (Figure 5A,B) confirmed the data. This proliferative activity in pericytes was also confirmed using immunofluorescence for PHH3 and αSMA in confocal microscopy (Figure 5C). Although the double immunochemistry procedure to explore nuclear Ki-67 expression was performed separately for each cell type (cytoplasmic expression of CD34 for ECs and αSMA for pericytes), the Ki-67 nuclear expression was also seen in the other cell type with unmarked cytoplasm, identifiable by its location (Figure 4D–F). Mitotic figures were only detected in pericytes of Type I BM (Figure 5D,E). Through the same procedures (double immunochemistry for Ki-67 and CD34 or αSMA) (Figure 5F), 50% of clusters and occasional garland-like structures (Type II BM) presented a PI of 1.12% ± 0.21% in pericytes and of 2.21% ± 0.88% in ECs. Therefore, the PI of pericytes in Type I BM was 37.59 times higher than in Type II BM. In some clusters, vessels with characteristics of Types I and II BM (transitional forms) were observed. These clusters were not used to calculate the PI.

### 3.6. Intravascular Pillars

By double staining for CD34 and SMA, two main types of intravascular pillars were observed depending on tissue components: (a) with extensions of CD34^+^ endothelium only, and (b) with a cover formed by CD34^+^ endothelium and a core formed by SMA^+^ pericytes.

**Figure 6:**
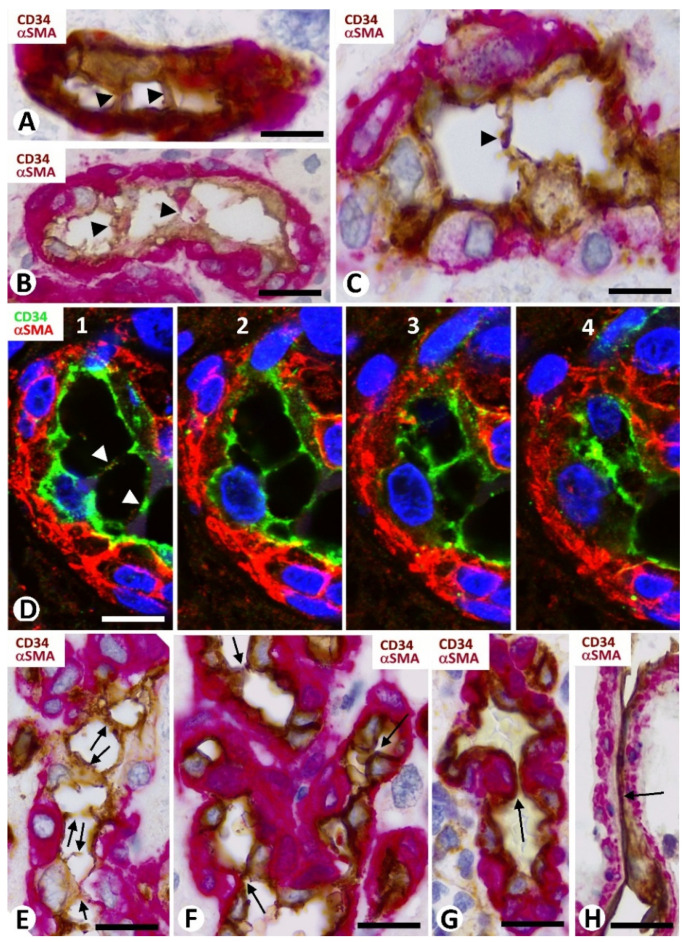
Pillars and EC contacts in bizarre vessels of GBM. (**A**–**E**) EC bridges (arrowheads and arrows) are observed between opposite vessel walls. Note in (**D1–4**) the appearance and disappearance of pillars in a series of individual views in confocal microscopy. Two or more pillars can be observed in the same vessel (**A**,**B**,**E**). (**F**–**H**) Apical (F, arrows)) and planar (**G**,**H**, arrows) EC contacts from opposite vessel walls. Observe that the planar contacts can be with (**G**) or without (**H**) prominence of pericytes (red). (**A**–**C**,**E**–**H**) Double immunochemistry for CD34 (brown) and αSMA (red). Hematoxylin counterstain. (**D1–4**): Double immunofluorescence for CD34 (green) and αSMA (red). DAPI counterstain. Bar: (**A**,**B**,**E**–**G**) 20 µm, (**C**,**D**) 15 µm, and (**H**) 40 µm.

In the first type of pillar, intraluminal bridges formed by CD34^+^ ECs and their processes were seen extending between opposite walls of small vessels that form the glomeruloid bodies and their precursor structures (Figure 6A–C). The pillars were thin, and their appearance and disappearance were detected in a series of individual views in confocal microscopy (Figure 6D1–4). Multiple pillars were present in some vessels (Figure 6E).

Contacts between ECs of opposite vessel walls were frequently observed (Figure 6F–H). These contacts were apical between prominent ECs (Figure 6F) or planar between flattened ECs (Figure 6G,H). The planar contacts could show prominence of the pericytes toward the vessel lumen (apparent push) (Figure 6G) or not (Figure 6H).

The characteristics, arrangement, and morphological development of the second type of pillar, which showed an endothelial cover and a pericytic core, depended on (a) vessel size, (b) formation of vessel loops and ITSs, as well as opening or not of the loop lumen surrounding the ITSs, and (c) pericyte hyperplasia and hypertrophy. Next, we describe the variants of this type of pillar depending on the conditions outlined above.

**Figure 7:**
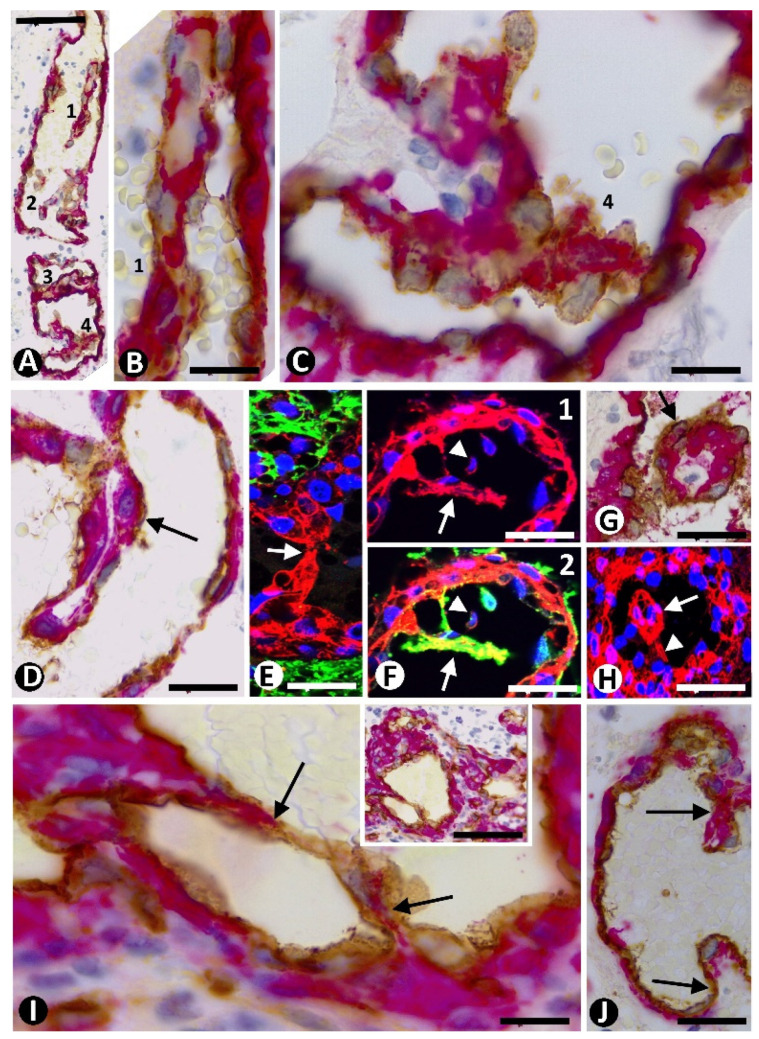
Pillars with an endothelial cover and a pericytic core in dilated vessels of GBM. (**A**) Pillars (1–4) are observed in a vessel. (**B**,**C**) Pillars 1 and 4 at higher magnification. (**D**) A pillar (arrow) presenting a core with αSMA^+^ pericytes (red) and a cover formed by CD34^+^ ECs (brown). (**E**–**H**) Characteristics of the pillars in longitudinal and cross sections (arrows). Note in (**F**) and (**H**) that thicker pillars give rise to thinner pillars (arrowhead). (**I**) Detailed view of a pillar in which processes of pericytes are only perceptible in the ends of the pillars (arrows) (insert at lower magnification). (**J**) Pillars (arrows) originating from two opposite points of the vessel wall. (**A**–**D**,**G**,**I**,**J**) Double immunochemistry for CD34 (brown) and αSMA (red). Hematoxylin counterstain. (**E**) Double immunofluorescence for GFAP (green) and αSMA (red). DAPI counterstain. (**F1**,**H**): Immunofluorescence for αSMA (red). DAPI counterstain. (**F2**) Double immunofluorescence for CD34 (green) and αSMA (red). DAPI counterstain. Bar: (**A**,**E**) 45 µm, (**B**,**C**,**I**) 15 µm, (**D**) 30 µm, (**E**) 45 µm, and (**F**–**H**, insert of **I**,**J**) 40 µm.

In dilated vessels (including some in glomeruloid bodies), pillars could be observed with a different extension and alignment in the vessel lumen (Figure 7A–J). When the pillars were longitudinal or cross-sectioned, the ECs formed the pillar cover and the pericytes formed the core (ITS) (Figure 7D–H). The cells in the pillar core ranged between thin processes of αSMA + pericytes perceptible in the ends of the pillar (Figure 7I) to rows of pericytes along it (Figure 7D). On occasion, pillars were seen originating simultaneously from two opposite points of the vessel wall (Figure 7J).

**Figure 8:**
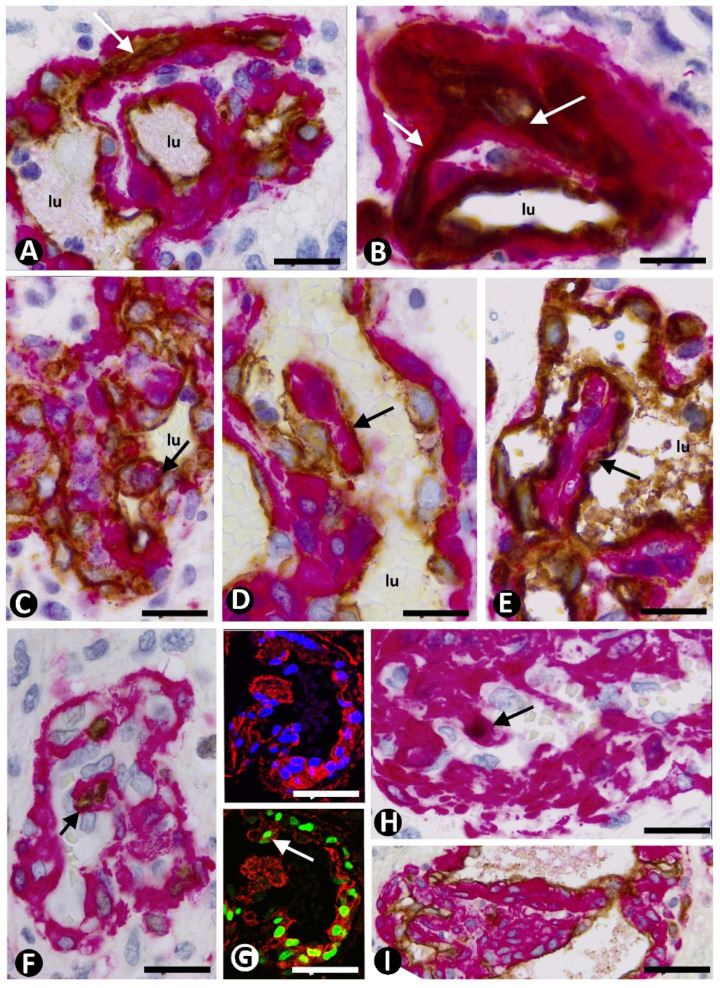
Loops, pillars, and pericyte proliferation in BM of GBM. (**A**–**E**) Vessel loops with virtual (**A**,**B**, arrows) or partially opened (**C**–**E**) lumen are observed originating from mother vessels or other loops. Note that the loops with virtual lumen (**A**,**B**, arrows) surround ITSs (αSMA^+^ pericytes, red). The loops with partially opened lumen form pillars, which are observed cross- (**C**, arrow), obliquely, or longitudinally sectioned (**D**,**E**, arrows). (**F**–**I**) Pericytes expressing Ki-67 and PHH3 in the core of pillars (**F**–**H**, arrows) and widening of the pillar core (**I**). (**A**–**E**,**I**) Double immunochemistry for CD34 (brown) and αSMA (red). Hematoxylin counterstain. (**F**,**H**) Double immunochemistry for Ki-67 (brown) and αSMA (red). Hematoxylin counterstain. (**G**) Immunofluorescence for αSMA (1) and double immunofluorescence for PHH3 (green) and αSMA (red) (2). DAPI counterstain. Bar: (**A**–**F**) 20 µm, (**G**) 45 µm, (**H**) 40 µm, and (**I**) 30 µm.

The relationship of pillars with vessel loops and ITSs was a common finding. Vessel loops were observed originating from mother vessels or other loops in the glomeruloid bodies and precursor structures (Figure 8A,B). Each fully formed vessel loop presented a virtual or open lumen, and its internal EC layer surrounded an ITS made up by αSMA^+^ pericytes (Figure 8B). In the glomeruloid bodies and precursor structures, the vessel loops and ITSs formed part of a conglomerate of similar structures when the vessel loop lumen was virtual (Figure 8C). When some of the vessel loop lumen was open, the ITS and its surrounding ECs formed the core and cover of a pillar, respectively (Figure 8D,E).

Expression of Ki-67 and PHH3 was observed in the αSMA pericytes surrounded by the loops without open lumen and in those present in the pillar cores (Figure 8F,G), which were widened, contributing to the formation of true pericyte aggregates in glomeruloid bodies and precursor structures (Figure 8I).

## 4. Discussion

In GBM, we studied the morphogenesis of the BM (clusters, garland-like structures, and glomeruloid bodies) and report as main findings (a) disproportion in the pericyte/EC proliferation (high proliferative activity of pericytes in relation to ECs), and (b) several mechanisms of intussusceptive angiogenesis. Next, we take into consideration (a) the vascular patterns in GBM, (b) the high proliferation of pericytes in relation to ECs, leading to pericyte hyperplasia and hypertrophy, and extension and thinning of ECs, and (c) the mechanisms of pillar formation (mechanisms of intussusceptive angiogenesis) and their participation in bizarre vessel morphogenesis.

In the classification of the main microvascular patterns in GBM, we followed the previous systematizations of other authors [1,2,3,4,5]. Thin-walled vessels are considered an exponent of classic microvascular sprouting (“classic vascular pattern”), while the other patterns (cluster/garland-like/glomeruloid bodies) are considered bizarre (malformed/aberrant) microvasculature, which was the object of our study. Given the observation of multiple transitional structures in the BM, we encompassed them as glomeruloid bodies and precursor structures. In the classification of GBM microvasculature, some authors grouped the patterns into two types depending on the prognostic significance. Thus, microvascular sprouting and vascular clusters were considered as Type 1 and vascular garlands, glomeruloid bodies, and vascular mimicry were considered as Type 2, with a more negative influence on progression-free survival in the latter [3]. The patterns in this classification partially coincide with those in the two types of our study in BM, based on the characteristics of the pericytes (patterns with vessels of thin walls and signs of sprouting, and vascular mimicry were not included in our study). Based on pericytic characteristics, our classification of BM facilitated PI comparison between different BM structures, including the PI between pericytes and ECs, demonstrating important imbalances. The classification, therefore, allowed us to respond to one of the approaches to BM formation. Given that our objective was focused on and limited to the mechanisms of BM formation, future studies with more extensive data are required for the clinical meaning and therapeutic implications of these mechanisms, above all in the response to antiangiogenic treatment.

Our observations revealed that the high PI in the BM corresponds mostly to prominent αSMA^+^ pericytes, to the extent that there is a manifest disproportion between the pericyte and EC PI (47.85 times higher in pericytes than in ECs). This high PI in pericytes concurs with the activation of endogenous pericytes in response to orthotopic mouse gliomas [33]. Abundant pericytes/vascular smooth muscle cells, a heterogeneous population [34,35] (e.g., the expression or not of MSA), have been previously observed in the microvasculature of glioblastoma [5,36,37]. This behavior of pericytes also contributes to the heterogeneity of angiogenesis and blood vessel maturation in human tumors [38,39]. The marked hyperplasia and hypertrophy of pericytes, which occupied the entire EC abluminal surface of the frequently folded bizarre vessels, suggest that their contractile force can be an important factor in vessel folding and contraction (e.g., capillaries with tortuous and narrow lumen in glomeruloid bodies). Likewise, the great proliferation of pericytes in the BM has morphogenic and clinical interest since pericytes facilitate (a) GBM growth through immunosuppression (reduction in T-cell activation) and anti-inflammatory response, and (b) GBM cell resistance to temozolomide or chemotherapeutic efficacy by targeting glioma stem cell-derived pericytes, which disrupt the blood–tumor barrier [40,41,42,43,44,45]. Likewise, the inhibition of NG2 proteoglycan, which promotes tumor vascularization [46] and is expressed by pericytes, induces a decrease in GBM microvessels [47,48]. The presence of structures of BM with different pericyte PI (PI of pericytes in Type I BM was 37.59 times higher than in Type II BM) and of transitional forms also suggests successive changes in preexisting pericytes, including the emergence of high proliferative activity. Further studies are required on the factors that participate in the different proliferative response of pericytes and ECs in the BM of GBM.

The low PI in ECs of the BM also suggests that they form vessel loops mainly by migration and extension in this aberrant type of microvascularization. A similar EC extension has been described in intussusceptive angiogenesis by vessel loop formation in the ovarian pedicle after ovariectomy and human tumor xenograft [25,26]. Although the PI in ECs is low with respect to that of pericytes, it confirms proliferation and survival of ECs in BM [49].

Iba1-positive microglia/tumor-associated macrophages (TAMs)/glioma-infiltrating macrophages (GIMs) around vessels with an amoeboid appearance suggest a perivascular reactive response, secondary to the lesion.

The presence of intravascular pillars, the hallmark of intussusceptive angiogenesis, in BM was confirmed by their appearance and disappearance in successive serial sections in confocal microscopy. The precursor findings involved in pillar morphogenesis in the BM of GBM coincide with one of the main mechanisms proposed in classical studies on vessel intussusception [20,21,22,23,24,25,26,27,50]. Thus, in vessels with a small lumen, intraluminal endothelial bridges appeared between the opposite vessel walls formed by thin EC filopodial ridges. Our observation of numerous apical and planar contacts of ECs also concurs with the participation of these structures in the initial phase of pillar formation.

A core formed by pericytes, stromal cells, and extracellular matrix, and a cover formed by ECs have been described in well-developed pillars. The only cells present in the pillar core in the bizarre vessels of GBM, mainly in those with a wider lumen, were αSMA^+^ pericytes. The observation of thin processes of αSMA^+^ pericytes between ECs in the pillar ends coincides with evolutive stages II and III of pillar formation described as a bilayer EC arrangement and pillar perforation [49].

Vessel loops have been demonstrated to contribute to pillar formation in the microvasculature [25,26,29,51,52]. Our observations in the BM of GBM reveal that vessel loops form part of an important mechanism of morphogenesis of these vessels by intussusceptive angiogenesis. Indeed, each loop surrounds the pericytes located between it and its mother vessel. These incarcerated pericytes make up an ITS. When the loop lumen is open, the EC sheets that form the internal endothelial layer of the loop and the ITS (encircled pericytes) are partially incorporated into the loop lumen, which is connected from two points (the two connecting segments of the loop) with that of the mother vessel. Thus, the surrounding endothelium and the ITS contribute to forming the pillar cover and core, respectively. While the pillar was intraluminal, the ends appeared connected to the wall of the mother vessel. When the loop lumen remains closed, ECs and pericytes form a solid set. Importantly, pericytes in the core of pillars also showed high proliferative activity, which increased pillar core size.

The contribution of mechanisms of intussusceptive angiogenesis to the BM of GBM is also of clinical interest. GBM is a highly malignant neoplasia, in which GBM angiogenesis and chemotherapy may be conditioned by multiple factors, including tumor location and natural substances [53,54,55]. In addition, GBM is difficult to treat effectively for lengthy survival, with many facets of therapy resistance and tumor recurrence [56,57,58]. Among these difficulties is the resistance to antiangiogenic therapy, in which the switch from sprouting to intussusceptive angiogenesis has been described as an escape mechanism [59].

**Figure 9:**
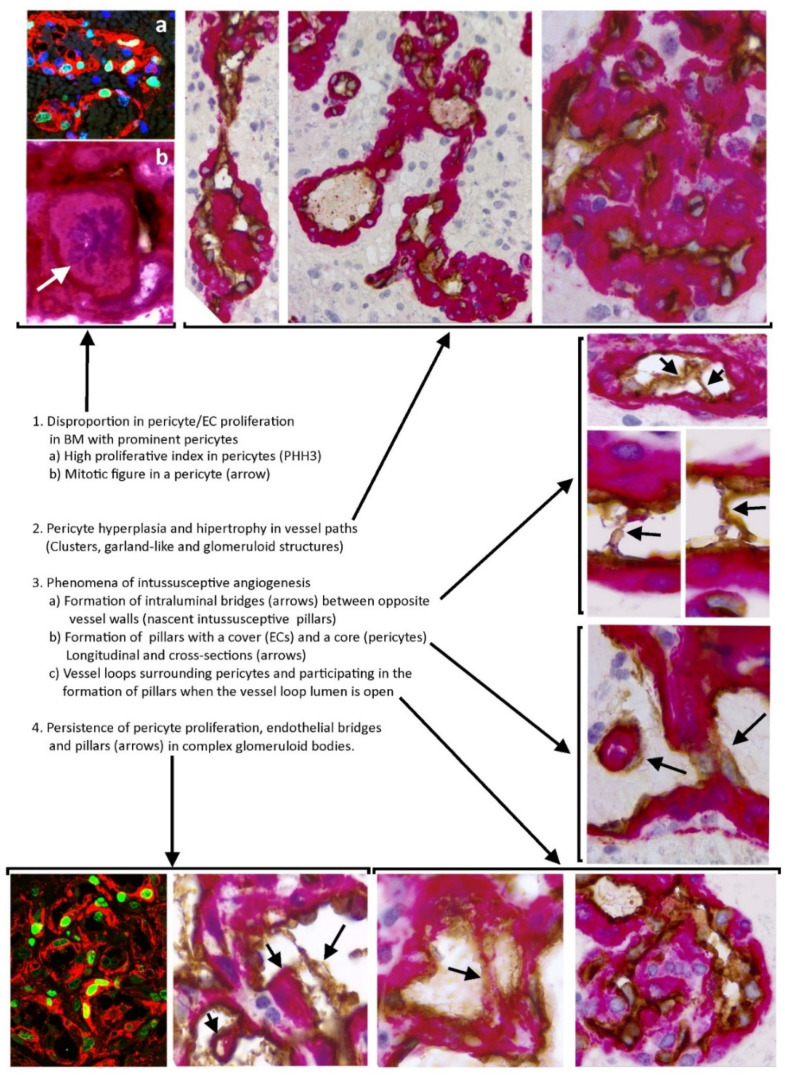
Summary with images of the mechanisms involved in bizarre microvasculature formation in GB.

In conclusion, the facts leading to the formation of the BM in GBM can be summarized as follows (Figure 9): (1) disproportion in pericyte/EC proliferation, highly in favor of pericytes, (2) pericyte hyperplasia and hypertrophy in most of MB, (3) phenomena of intussusceptive angiogenesis, including (a) intraluminal endothelial bridges (intussusceptive microvascular growth), (b) development of intussusceptive pillars with a cover (ECs) and a core (pericytes), and (c) formation of numerous vessel loops, which also participate in pillar development when the loop lumen is open and their internal ECs surround an ITS (pericytes), and (4) persistence of these facts in the most evolved structures of the BM, such as complex glomeruloid bodies. Therefore, a disproportion in the pericyte/EC PI (with high pericyte proliferation) and mechanisms of intussusceptive angiogenesis participate in BM formation in GBM. In addition to the improved knowledge of the mechanisms that participate in the formation of the BM in GBM, these contributions are of clinical interest, since pericytes and intussusceptive angiogenesis can condition antiangiogenic therapy.

## Figures and Tables

**Table 1:** Proliferation Index (PI) in bizarre microvasculature (BM) of GBM.

	Type I BM	Type II BM
**Overall PI**	Mean 43.57 ± 9.5	Mean 3.41 ± 0.76
**Pericyte PI**	Mean 42.11 ± 5.76	Mean 1.12 ± 0.21
**EC PI**	Mean 0.88 ± 0.29	Mean 2.21 ± 0.88

Type I BM: Prominent, plump pericytes covering all EC abluminal surface (glomeruloid bodies, most of vascular garlands and ~50% of vascular clusters). Type II BM: Elongated, branched pericytes partially covering the EC abluminal surface (~50% vascular clusters and occasional vascular garlands). Pericyte PI/EC I in Type I BM: 42.11 (±5.76)/0.88 (±0.29). Pericyte PI in Type I BM/ Pericyte PI in Type II BM. 42.11 (±5.76)/1.12 (±0.21). *p* < 0.002. PI in Type I BM 47.85 times higher in pericytes than in ECs. PI in pericytes in Type I BM 37.59 higher than in Type II BM.

## Data Availability

All the data are reported in the present paper.
